# Improve Temporal Fourier Transform Profilometry for Complex Dynamic Three-Dimensional Shape Measurement

**DOI:** 10.3390/s20071808

**Published:** 2020-03-25

**Authors:** Yihang Liu, Qican Zhang, Haihua Zhang, Zhoujie Wu, Wenjing Chen

**Affiliations:** College of Electronics and Information Engineering, Sichuan University, Chengdu 610065, China; lyh@stu.scu.edu.cn (Y.L.); haihua@zjnu.cn (H.Z.); zhoujie_wu@163.com (Z.W.); chenwj0409@scu.edu.cn (W.C.)

**Keywords:** high-speed 3-D measurement, dynamic scene measurement, fringe projection, mechanical projector, Temporal Fourier Transform Profilometry (TFTP), phase unwrapping

## Abstract

The high-speed three-dimensional (3-D) shape measurement technique has become more and more popular recently, because of the strong demand for dynamic scene measurement. The single-shot nature of Fourier Transform Profilometry (FTP) makes it highly suitable for the 3-D shape measurement of dynamic scenes. However, due to the band-pass filter, FTP method has limitations for measuring objects with sharp edges, abrupt change or non-uniform reflectivity. In this paper, an improved Temporal Fourier Transform Profilometry (TFTP) algorithm combined with the 3-D phase unwrapping algorithm based on a reference plane is presented, and the measurement of one deformed fringe pattern producing a new 3-D shape of an isolated abrupt objects has been achieved. Improved TFTP method avoids band-pass filter in spatial domain and unwraps 3-D phase distribution along the temporal axis based on the reference plane. The high-frequency information of the measured object can be well preserved, and each pixel is processed separately. Experiments verify that our method can be well applied to a dynamic 3-D shape measurement with isolated, sharp edges or abrupt change. A high-speed and low-cost structured light pattern sequence projection has also been presented, it is capable of projection frequencies in the kHz level. Using the proposed 3-D shape measurement algorithm with the self-made mechanical projector, we demonstrated dynamic 3-D reconstruction with a rate of 297 Hz, which is mainly limited by the speed of the camera.

## 1. Introduction

Optical non-contact three-dimensional (3-D) shape measurement technique has been widely used in science, industrial applications and our daily life, due to its high resolution, high-speed, and high flexibility. Among others, fringe projection profilometry approaches have proven to be the most promising techniques [1,2]. According to the different approaches of phase retrieval, fringe projection profilometry can be divided into Phase Measurement Profilometry (PMP) [3,4,5,6], Fourier Transform Profilometry (FTP) [7,8], Wavelet Transform Profilometry (WTP) [9,10], Windowed Fourier Transform Profilometry (WFTP) [11], and so on. These fringe projection techniques extract the phase corresponding to the object’s height by employing an arctangent calculation. Therefore, the phase distribution is wrapped to the principle value ranging between − π and π, consequently, the phase unwrapping must be carried out to obtain a continuous phase map. The commonly used phase unwrapping algorithms can be divided into two categories, spatial phase unwrapping [12,13,14] and temporal phase unwrapping [15,16,17]. For spatial phase unwrapping, only a single wrapped phase map is employed and the unwrapped phase of a measured object is derived. However, such spatial phase unwrapping algorithms have one common limitation: they tend to fail when the wrapped phase contains discontinuous or isolated parts [16]. For temporal phase unwrapping, they can be used to analyze the highly discontinuous object, because each spatial pixel from the measured data is unwrapped independently [15]. The common feature of temporal phase unwrapping is that they need more than one unwrapped phase distribution or some additional coded patterns to provide extra information about the fringe orders [18]. 

In the past few years, along with the increased demands on dynamic scene measurement, the high-speed 3-D shape measurement has been a key research problem [19]. In particular, dynamically moving or deforming objects are to be measured. For the 3-D dynamic shape measurement process, while using a series of N (N ≥ 1) patterns, the object should be kept stationary during the projection of N fringe patterns. If the measurement object and the sensor system move relative to each other, an additional unknown phase-shifting in the captured images will occur, resulting in motion-induced phase error and thus depth measurement error. There have been several approaches to improve measurement accuracy for dynamic measurement. Firstly, some scholars developed methods to compensate for errors caused by object relative motion during measurement [20,21]. While these motion-induced-error compensation methods work well, they usually require extra computational cost and additional processing to suppress errors at fringe edges. Secondly, single-shot technique (N = 1), such as FTP using spatial phase unwrapping, is a popular and efficient way. However, due to the band-pass filtering, FTP method has trouble measuring an object with sharp edges, abrupt change or non-uniform reflectivity. In last paper, we proposed a new phase extraction method, Temporal Fourier Transform Profilometry (TFTP) [22]. Compared to FTP, TFTP needs no weighted filtering operation in the spatial domain, and the spatial high-frequency components of the measured object have been successfully retained. Nevertheless, in order to make sure that the 3-D unwrapped phase distribution calculated by TFTP can be wrapped correctly in a whole 3-D space, one of the wrapped phases must be successfully unwrapped in 2-D spatial domain. This condition prevents the TFTP method from being used to measure the absolute isolated objects. However, with the wide application of fringe projection profilometry, complex objects such as isolated or abrupt objects often exist in the current application scenes, which is inevitable. For this reason, in this paper, 3-D phase unwrapping method based on the reference plane is presented to realize phase unwrapping of the 3-D wrapped phase of an absolute isolated complex object. Combined with the TFTP method, we achieved 3-D shape measurement of an isolated abrupt object. In addition, one deformed fringe pattern can produce a new reconstruction result, which avoids motion-induced-error in dynamic the measurement process. Experiments have been performed to verify the performance of the proposed method.

In addition, other scholars reduced the effect of object motion during measurement by improving the projector. In contrast to the camera with high sensitivity, high frame rate, and high resolution, which have been commercially available for some time, the most commonly used high-speed projection device, such as liquid crystal display (LCD) or digital light processing (DLP) projector, can only operate properly within a limited spectrum light range and are restricted in terms of speed (especially in 8-bit greyscale mode). To overcome the limitation of DLP techniques, Patrick et al. developed a set of high-speed sinusoidal structured light illumination systems in 2011 [23], the 3-D surface was reconstructed by the coded phase shift (CPS) method. They achieved a binary pattern projection at the rate of 200 Hz and the 3-D reconstruction rate of 20 Hz. However, in their method, the strict mechanical error analyzing and compensation strategies were required to obtain accurate a phase-shifting fringe pattern and the pattern could not be changed flexibly. From 2016 to 2018, Heist et al. proposed and optimized GOBO (GOes Before Optics) projector, which used a rotating slide structure to project aperiodic sinusoidal fringe patterns at high frame rates and with a high radiant flux of 250 W [24,25]. They achieved a 3-D frame up to 50 KHz. While, due to the unknown position of the GOBO wheel and the feature of aperiodic sinusoidal fringe project method, an extra high-speed camera was required for 3-D measurement. At the same time, Hyun et al. [26] also developed a 3-D measurement system that consisted of a mechanical projector and two cameras in 2018. They used encoded texture images and a disparity map between two cameras to reconstruct 3-D shape, and achieved 10,000 Hz 3-D shape measurement speed. Despite both the above methods having good performances, these techniques use two cameras and one projector, which will produce more shadow-related problems since all three devices must see the same point in order to measure its height. Moreover, the high-speed cameras are usually very expensive. Therefore, using a single projector and a single camera for high-speed measurements is still desirable and has its practical application requirements. 

Inspired by the work of Heist [24,25], while different, in this paper, we use a self-made mechanical projector and one high-speed camera to form a low-cost and flexible dynamic 3-D measurement equipment. The uniform radial fringes of the rotating disc are used to generate the structural light field. Meanwhile, the signal of the rotating disc is strictly detected, and the camera is precisely synchronized with the projector by the signals to record phase-shifting fringe patterns for phase extraction. In addition, the disc of the projected pattern is easy to replace, which can be adjusted according to different measuring scenes. 

This paper is arranged as following: Section 2 illustrates the principle and the equipment of the proposed method exactly. Section 3 presents the experimental results to verify the performance of the proposed method. Section 4 discusses the strengths and weaknesses of the proposed method. Section 5 summarizes this work.

## 2. Principle and Equipment

### 2.1. Temporal Fourier Transform Profilometry Method

For a TFTP system, the projector produces a multi-period phase-shifting sinusoidal fringe on the surface of the object being measured. The imaging components synchronously record the deformation sinusoidal fringe modulated by the object. For a measurement process, at time *t*, the deformed fringe can be expressed as:

(1) $$g\left(x,y,t\right)=a\left(x,y\right)+b\left(x,y\right)cos(2\pi x/p_{0}+\varphi \left(x,y,t\right)+2\pi f_{t}t)$$

where *p*_0_ is the spatial period of the projected grating; φ(x,y,t) is the phase distribution modulated by the height information of the tested object at time *t*; *2πf_t_t* is the phase modulation at time *t* caused by the multi-period phase-shifting, and *f_t_* is the temporal period (or frequency) of the phase-shifting fringes. 

For easier description, Equation (1) can also be expressed by:

(2) $$g\left(x,y,t\right)=a\left(x,y\right)+\frac{b\left(x,y\right)}{2}e^{i2\pi f_{0}x}e^{i\varphi \left(x,y,t\right)}e^{i2\pi f_{t}t}+\frac{b\left(x,y\right)}{2}e^{-i2\pi f_{0}x}e^{-i\varphi \left(x,y,t\right)}e^{-i2\pi f_{t}t}$$

where *f_0_* is the spatial frequency of the projected fringe. For each point (*x, y*), the $e^{i2\pi f_{0}x}$ and $e^{-i2\pi f_{0}x}$ are a constant in the temporal domain. So, Equation (2) can be rewrote as:

(3) $$g\left(x,y,t\right)=a\left(x,y\right)+\frac{c\left(x,y\right)}{2}e^{i\varphi \left(x,y,t\right)}e^{i2\pi f_{t}t}+\frac{c’\left(x,y\right)}{2}e^{-i\varphi \left(x,y,t\right)}e^{-i2\pi f_{t}t}$$

Then 1-D Fourier transform is carried out along the temporal domain for each pixel’s signal; the frequency components of its Fourier spectrum *G* (*x, y, f*) can be separated and noted as:

(4) $$G\left(x,y,f\right)=a\left(x,y\right)\cdot \delta \left(f\right)+\frac{c\left(x,y\right)}{2}\psi \left(x,y,f-f_{t}\right)+\frac{c’\left(x,y\right)}{2}\psi^{*}\left(x,y,-\left(f+f_{t}\right)\right)$$

With a suitable filter window, the spectra are filtered to reserve only the fundamental component. The 1-D inverse Fourier transform *F*^−1^ is applied to the fundamental component, we obtain a complex signal:

(5) $$\hat{g}\left(x,y,t\right)=F^{-1}\left\{\frac{C\left(x,y\right)}{2}\psi \left(x,y,f-f_{t}\right)\right\}=\frac{C\left(x,y\right)}{2}e^{i\varphi \left(x,y,T\right)}e^{i2\pi f_{t}T}=\frac{C\left(x,y\right)}{2}e^{i\left[\varphi \left(x,y,t\right)+2\pi f_{t}t\right]}$$

Then, φ(x,y,t) can be extracted. After unwrapping in 3-D space, the continuous natural phase distribution Φ(x,y,t) can be obtained as:

(6) Φ(x,y,t)=unwrap{φ(x,y,t)}=unwrap{arctanIm[g^(x,y,t)]Re[g^(x,y,t)]}−2πftt

where, unwrap {#} is an unwrapping operator. In order to get the natural phase of a tested object, the time modulated factor 2π*f_t_t* should be removed. The 3-D shape distribution *h*(*x, y, t*) can be calculated with the help of the system calibration parameters.

Through the above analysis, it can be learned that TFTP method avoids doing Fourier transform, bandpass filter, and inverse Fourier transform on 2-D spatial domain, which can effectively retain the high-frequency components of the tested object, so it can be well applied to extract the natural phase of a dynamic object with abrupt changes. These specialties of TFTP determine that it is especially suitable for multi-frame 3-D shape measurement of a complex object, which cannot be successfully digitalized by the traditional FTP in 2-D spatial fringe analysis or a dynamic object that will appear as a motion-error for the multi-frame fringe analysis method. However, it greatly limits the application of TFTP method that one of the wrapped phases must be successfully unwrapped in 2-D spatial domain in order to ensure the entire phase continuity in 3-D space. For example, two isolated or complex surface objects will be error prone during 2-D spatial phase unwrapping. For TFTP method, an error in the original unwrapped phase will pollute the whole 3-D phase distribution. 

### 2.2. Three-Dimensional Phase Unwrapping Based on Reference Plane

In order to make the TFTP method have a wider range of applications, for the above analysis of TFTP’s limitations, a new 3-D phase unwrapping method is presented in this section. A reference plane’s phase is used to assist the phase unwrapping process, making the TFTP method more accurate and robust in the measurement of the isolated objects. 

The improved TFTP algorithm proposed in this paper can be described in the following five steps:

(1) For the deformed fringe pattern captured by camera, 1-D Fourier transform, spectrum filtering and inverse Fourier transform are performed along the time axis on each pixel, getting the 3-D wrapped phase distribution data φ(x,y,t) of a measured dynamic object.
(2) Calculating the unwrapped phase $\phi_{0}$ of the reference plane measured on the same system.
(3) Choosing one wrapped phase $\varphi \left(x,y,t_{1}\right)$ of the tested object at sampling time *t*_1_, in which, the object’s corresponding height changes within a suitable range. And comparing 2π with the phase difference between $\phi_{0}$ and $\varphi \left(x,y,t_{1}\right)$ to get the multiple integer K for each pixel, which must satisfy the following condition
  (7) $$2\pi \times \left(K-1\right)<\phi_{0}-\varphi \left(x,y,t_{1}\right)<2\pi \times K$$
  In other words, K can be determined as
  (8) $$K\left(x,y\right)=ceil\left[\frac{\phi_{0}-\varphi \left(x,y,t_{1}\right)\;}{2\pi }\right]$$
  where, ceil [*] is the ceiling operator that gives the nearest upper integer number.
(4) Adding 2Kπ to $\varphi \left(x,y,t_{1}\right)$ to get its unwrapped phase $\phi \left(x,y,t_{1}\right)$
  (9) $$\phi \left(x,y,t_{1}\right)=\varphi \left(x,y,t_{1}\right)+2K\pi$$
(5) Taking $\phi \left(x,y,t_{1}\right)$ as the benchmark of the 3-D phase unwrapping and performing the 1-D phase unwrapping along the temporal axis, then finally obtaining the 3-D unwrapped phase distribution.

**Figure 1:**
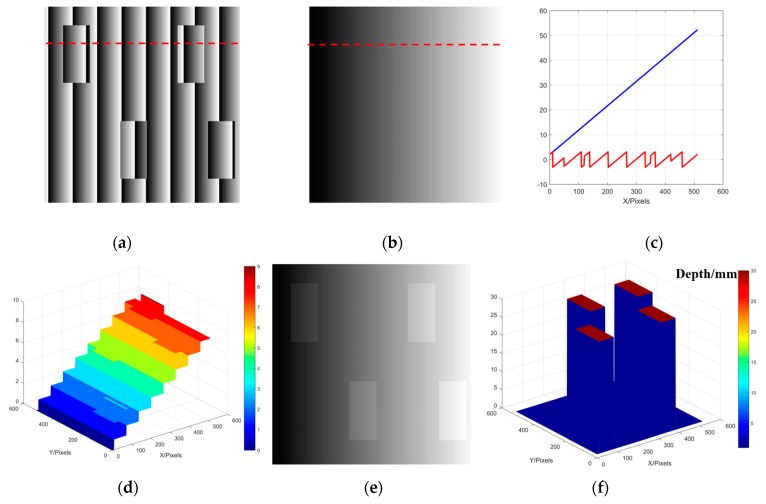
Schematic diagram of phase unwrapping based on reference plane; (**a**) Wrapped phase of the measured object; (**b**) Unwrapped phase of the reference plane; (**c**) 100th row of the wrapped phase and the unwrapped phase of the reference plane; (**d**) Value of the order K; (**e**) Unwrapped phase of the measured object; (**f**) Reconstructed result of measured objects.

The reference plane we used to assist phase unwrapping is one of the calibration planes. During the calibration process, only a limited number of planes (usually four planes are measured for the three unknown parameters calculation [27]) are obtained in the whole measurement depth range. After the determination of $\varphi \left(x,y,t_{1}\right)$, the calibration plane whose phase of its actual height is closest to $\varphi \left(x,y,t_{1}\right),$ is selected as the reference plane to ensure the wrapped phase can be unwrapped correctly. In addition, it is not excluded that sometimes other planes among the limited ones will be chosen to test whether it is more appropriate. To better illustrate the reference plane to guide the phase unwrapping, we simulate the phase unwrapping process of an isolated abrupt object. As shown in the Figure 1, Figure 1a–f are the phase unwrapping process of four cuboids isolated from each other, and all the heights of cuboids are 30 mm. Figure 1a is the wrapped phase, and Figure 1b is the unwrapped phase of the reference plane. Taking the data along 100th row as an example, the wrapped phase and the unwrapped phase of the reference plane are plotted in Figure 1c. Comparing 2*π* with the phase difference between the unwrapped phase of reference plane and the wrapped phase of the tested object, we can obtain the order *K* as shown in Figure 1d. Then adding 2*Kπ* to the wrapped phase, the correct unwrapped phase as shown in Figure 1e can be obtained. Figure 1f is the reconstruction result. Different from the spatial phase unwrapping, the reference plane is used to assist the phase unwrapping process. Each pixel is independently unwrapped by using the phase information of the reference plane, instead of the adjacent pixel’s phase information. Therefore, this method can successfully unwrap the selected 2-D wrapped phase distribution of an isolated measured object in 2-D spatial domain to make a benchmark for 3-D phase unwrapping.

### 2.3. High-speed Measurement System and Framework of the Proposed Method

Our experimental device is composed of a self-made mechanical projector and a high-speed camera (Baumer HXC40NIR, up to 300 fps @ 1024 × 1024 pixels). The mechanical projector contains the following components, a light source, Fresnel lens (for collimation and homogenization), a self-made grating disc, an optical synchronization unit, and a projection lens.

**Figure 2:**
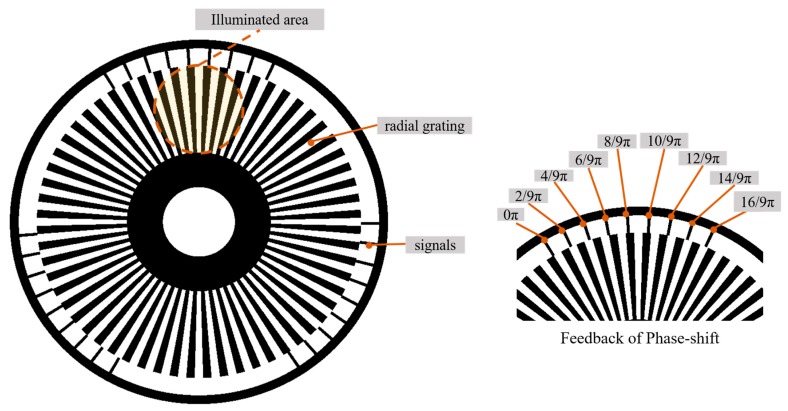
Schematic diagram of self-made grating disc design.

The self-made grating disc includes two parts, radial grating part and signals part, as shown in Figure 2. The radial part is a binary grating that will produce a sinusoidal fringe pattern by defocusing in the measurement process. The signal part of the disc can accurately feedback the phase-shifting information and transmit the square waves to synchronously control the used camera. The signal part used in this paper will track and feedback the nine-step phase-shifting signal. In addition, the various phase-shifting fringes can be obtained by resampling the square waves or redesigning the signal part.

**Figure 3:**
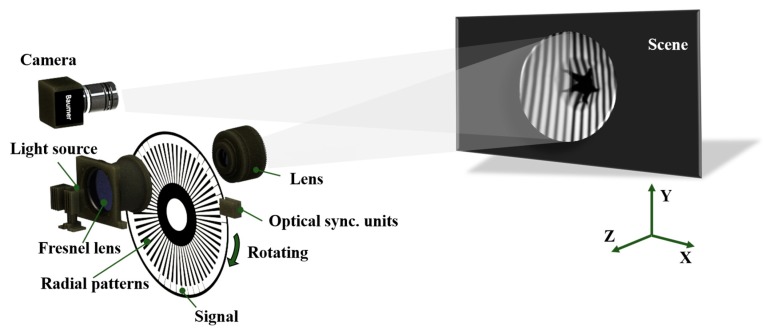
Schematic diagram of our proposed mechanical projection system.

The experimental device is shown in Figure 3. In our presented projection device, the light from the source is collimated and homogenized by Fresnel lens and then illuminated by the self-made grating disc, a series of fringe patterns that are transported by constant rotational motion (see Figure 3) and imaged onto an object to be measured. The signal part collects the phase-shifting information and generates the square wave, which is strictly synchronized with the high-speed camera. The speed of the rotational motion driven by the motor can be adjusted to a maximum of 3000 rpm (round per minute). The self-made grating disc used in this paper can produce 27 signals within one rotation. If the motor speed reaches 3000 rpm, our mechanical projector can reach thousands of projection frequency per second. Due to the limitation of the shooting speed of the used camera, the motor is rotated at 657 rpm in this paper. 

**Figure 4:**
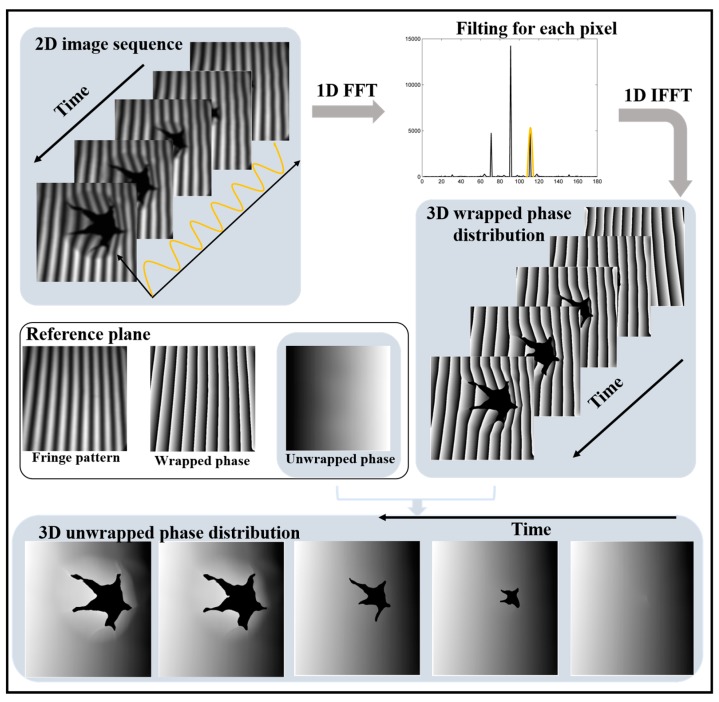
Computational framework of our proposed 3-D reconstruction method.

To illustrate the whole process of this proposed method clearly, the whole framework of this method is shown in Figure 4. During the measurement, the high-speed camera collects a series of deformation fringe patterns. After 1-D Fourier transform, filtering and inverse Fourier transform in temporal domain, 3-D wrapped phase distribution is obtained. With the reference plane unwrapped phase captured in advance, one wrapped phase of the whole 3-D wrapped phase distribution is unwrapped. The phase of this frame is taken as the benchmark while the 3-D wrapped phase distribution is unwrapped along temporal axis. In the whole process, each pixel is processed independently without using the adjacent pixels’ information in spatial domain, so this method can be successfully used for the measurement of the isolated and abrupt objects.

### 2.4. System Calibration

**Figure 5:**
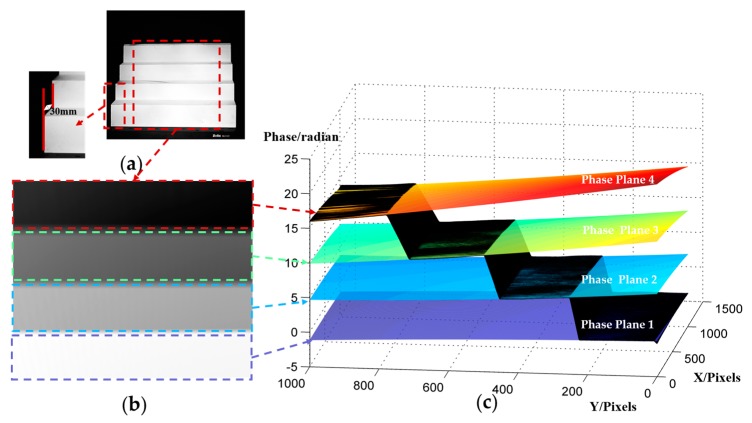
System calibration process: (**a**) Photograph of the standard block; (**b**) Absolute phase of the standard block; (**c**) Four fitted absolute phase planes based on the standard block’s absolute phase.

In order to obtain the 3-D surface information of the object, the system needs to be calibrated using the phase-to-height algorithm in advance [27]. Equation (10) can be used to reconstruct the height of a measured object.

(10) $$\frac{1}{h\left(x,y\right)}=u\left(x,y\right)+v\left(x,y\right)\frac{1}{\Delta \phi_{h}\left(x,y\right)}+w\left(x,y\right)\frac{1}{\Delta \phi_{h}^{2}\left(x,y\right)}$$

where Δ*ϕ_h_*(*x*, *y*) is the phase value of the measured object, relative to the reference plane. *h*(*x*, *y*) is the measured object’s height. Four planes with known height distributions should be measured for the three unknown parameters’ *u*(*x, y*), *v*(*x, y*) and *w*(*x, y*) calculation. In the usual measurement, we can use the stepper motor to drive a standard plane moving to the known positions of four planes. In this paper, the system was calibrated with a self-made standard block instead of four planes by moving. The self-made standard block, as shown in Figure 5a, is a step-like object whose four planes on the Z axis keep strictly parallel, with a distance of 30 mm between each plane. During the calibration process, the standard block is placed in the range of the calibrated field. After the absolute phase value of the standard block is obtained, the four full-filed phase distributions planes can be calculated through polynomials fitting, and the quadratic fitting is also used to reduce the effect of the imaging system’s aberration and distortion, as shown in Figure 5b,c.

## 3. Experiments and Results

### 3.1. Accuracy Analysis

**Figure 6:**
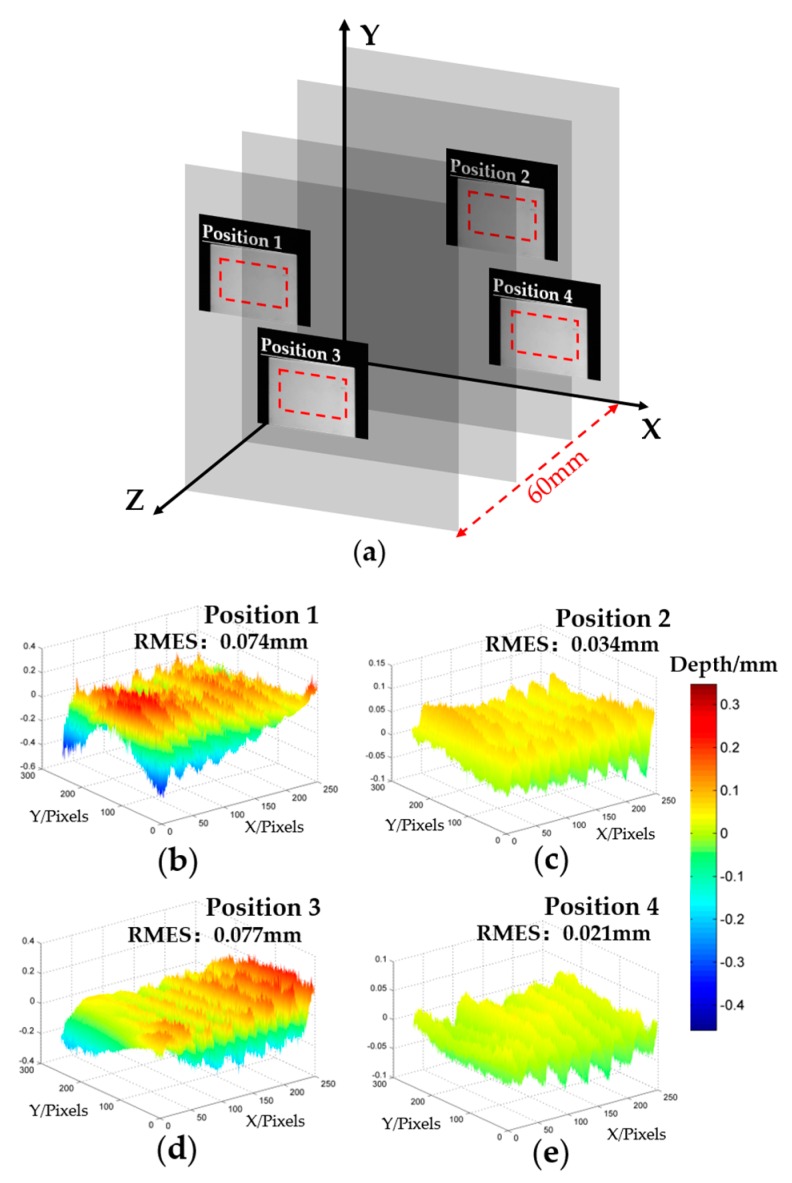
Accuracy analysis of our proposed method. (**a**) Four positions of the ceramic standard flat in the measured field; (**b**–**e**) Error distribution of standard flats of the four positions.

To quantify the accuracy of our system and this proposed method, a standard ceramic flat is measured at four positions as shown in Figure 6a; the depth range is 60 mm. The fitting plane based on the reconstructed result of the measured flat is used as the ground truth. The corresponding phase error distribution of the measured flat in four positions are shown in Figure 6b–e. The root-mean-square-error (RMSE) of the four positions is 0.074 mm, 0.034 mm, 0.077 mm, and 0.021 mm respectively. Our mechanical projector system obtains the sinusoidal fringe by defocusing binary structured patterns. For the binary defocusing technique, the degree of defocusing must be controlled to a certain range in order to produce high-quality fringe images [28]. The sinusoidal fringe quality will be affected by the defocusing degree. From the error analysis results, it can be seen that in the height range of position 2 and position 4, the error is smaller than the height range of position 1 and position 3 because of the suitable defocusing degree. The height range of Position 1 and Position 3 produced a larger error, due to the unsuitable defocusing degree.

### 3.2. Comparative Experiments on Isolated Objects

**Figure 7:**
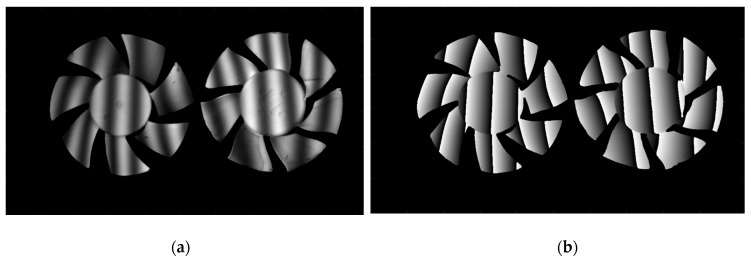

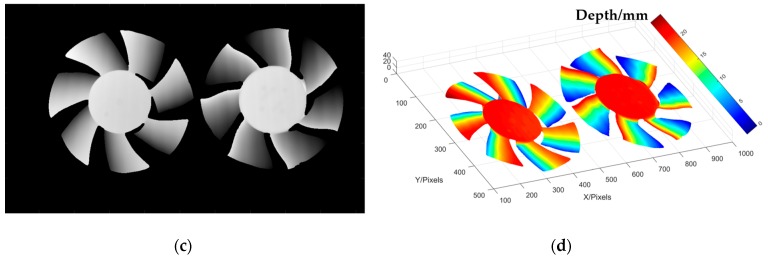
Measurement of the isolated objects by this proposed method. (**a**) One captured sinusoidal image of the measured scene; (**b**) Wrapped phase of the measured scene; (**c**) Absolute phase of the measured scene; (**d**) Reconstructed result of measured scene.

In order to verify the performance of the proposed method in the measurement of isolated and abrupt objects, two different fans isolated from each other are measured by our proposed method. One of the deformed sinusoidal fringes is shown in Figure 7a. Figure 7b shows the wrapped phase of the measured scene. Figure 7c shows their absolute phase, and Figure 7d shows the reconstructed result. For the improved TFTP method, the calculation is carried out pixel-to-pixel in the whole process, some pixels that the information loss or poor-quality will not lead to the error in either phase extraction or phase unwrapping process, and both the correct wrapped and unwrapped phase can be obtained. From these, it can be seen that our proposed method can well restore the 3-D shape of such isolated abrupt objects.

**Figure 8:**
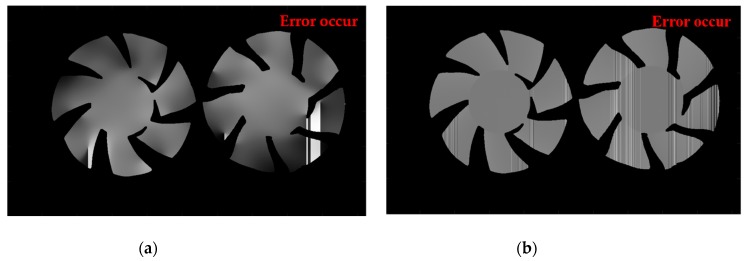

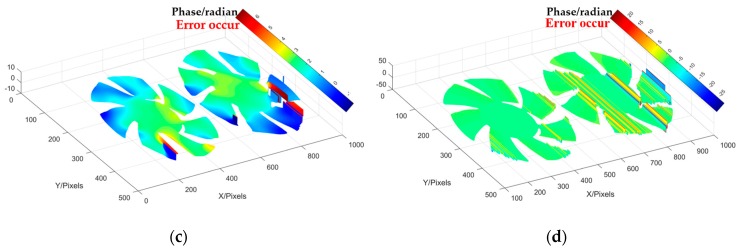
Comparative experiments about isolated object; (**a**) False unwrapped phase by FTP; (**b**) False unwrapped phase traditional TFTP; (**c**) Three-dimensional diagram of false unwrapped phase by FTP; (**d**) Three-dimensional diagram of false unwrapped phase traditional TFTP.

To further illustrate the advantages of our method over other methods, the FTP method and traditional TFTP method (proposed in [23]) have been used to measure the same scene for comparison. The experimental results are shown in Figure 8. From the phase reconstruction results, as shown in Figure 8a,b, we can see that for the absolute isolated measured objects in spatial domain, neither FTP nor TFTP can achieve 3-D reconstruction. The obvious error can be seen from the side view, shown in Figure 8c,d. For the FTP method, the measured object is steep, isolated and shadowed, all these factors will cause the spectrum overlapping and lead to the failure in extracting the spectral fundamental component, which contains the object’s height information. When the phase extraction fails, the wrapped phase will also be influenced. For the TFTP method, although the correct wrapped phase can be obtained successfully, because of the poor-quality phase information or the lack of phase information in some pixels, the spatial phase unwrapping method will fail in these pixels and propagate, causing line error. 

By comparing the results of FTP, traditional TFTP and improved TFTP, the data indicates that the TFTP method adopts pixel-independent processing during phase extraction and greatly reduces the error rate of the fundamental frequency information extraction. Therefore, it is better to use TFTP to obtain the phase information of spatially isolated objects. The improved TFTP algorithm further realizes the individual processing of pixels in the phase unwrapping process, which avoids the possibility of errors using spatial phase unwrapping about isolated objects.

### 3.3. Measurement of an Impact Process

**Figure 9:**
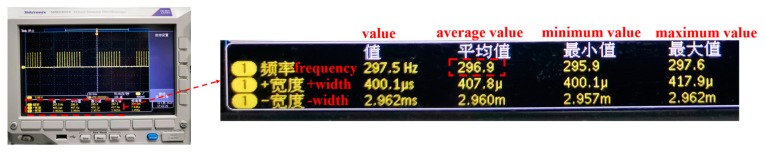
Pulse frequency captured by oscilloscope

**Figure 10:**
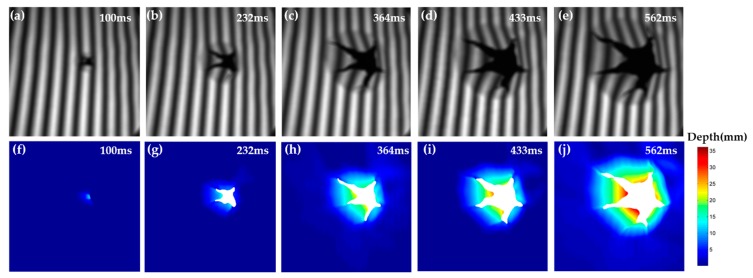
Measurement on the impact process scene. (**a**–**e**) Sinusoidal image of representative impact process; (**f**–**j**) 3-D reconstructions at the corresponding moments (Visualization 1).

In this experiment, an impact process was measured to prove the feasibility of this proposed method in dynamic 3-D measurement. A cracking sheet under the rapid impact of a flying hammer within 1 s was targeted, and there was abrupt and fringe information missing during the sheet cracking. In this experiment, according to the measured scene changing speed, the rotation speed of the mechanical projector was adjusted to 657 rpm. The mechanical projector sends out 27 pulses per rotation to feedback the phase-shifting information. Figure 9 shows the pulse frequency, which is displayed by the oscilloscope during the measuring process. The average value is 296.9 Hz. In the whole process, the Baumer HXC40NIR camera captures 300 frames. Some of the fringe patterns, 512 × 512 pixels, recorded at 100 ms, 232 ms, 364 ms, 433 ms, and 562 ms respectively are shown in Figure 10a–e and their corresponding 3-D reconstruction results are shown in Figure 10f–i. The reconstruction rate is 297 fps. The associated Visualization 1 displays the impact process. There is no need to bass-filter in the spatial domain, and pixel-to-pixel processing makes it possible to reconstruct 3-D information of a dynamic object, which has abrupt or partial fringe information missing. Experimental results show that the proposed method has good performance for high-speed scenarios with abrupt and rapid changes.

## 4. Discussion

The major difference between our proposed method and that developed by Heist in Refs. [25,26] are listed to better distinguish: (1) our self-made disc consists of two parts, the grating part and the signal part. The grating part is the binary periodic radial fringe, and the periodic sinusoidal fringe pattern is obtained by defocusing during the measurement. (2) The signal part can accurately feedback the phase-shifting information, and the camera gets an accurate phase-shifting fringe through the synchronization component. (3) Our device consists of a self-made mechanical projector and a single high-speed camera, and the phase of the deformed periodic sinusoidal fringe is extracted by TFTP method for height recovery. (4) The reference plane’s phase information is used in the 3-D phase unwrapping to realize 3-D shape reconstruction the isolated object in the dynamic measured scene. 

Besides, the devices and methods we present in this paper have the following features compared with other high-speed 3-D shape measurement techniques.

- **A new absolute phase of an isolated, steep object can be recovered from a new distorted sinusoidal fringe pattern.** For a dynamic measured scene, compared with FTP, TFTP can also get new height information from each new distorted fringe pattern, and the motion error is avoided. Moreover, TFTP does not filter in the spatial domain but in the temporal domain, avoiding the spectral overlapping caused by the information loss of some pixels in the spatial domain and the smoothing effect of spatial Fourier regarding steep objects. The absolute phase recovery, pixel-by-pixel, is realized by introducing the unwrapping phase of the reference plane and unwrapping the 3-D wrapped phase distribution along the temporal axis. The difference is, for the TFTP method, data processing operations should be carried out after all the deformed fringe patterns are acquired.
- **TFTP have a better performance in the dynamic measured scene.** In the improved TFTP method, a new 3-D reconstruction result can be obtained from each new deformed fringe pattern. Compared with the PMP method and temporal phase unwrapping method, the improved TFTP will not cause a motion error in high-speed measurement. Nevertheless, in the TFTP method it is expected that the sampling theorem must be satisfied in the temporal domain because of the use of Fourier fringe analysis along the temporal axis (at least four sampling points per period to avoid spectrum overlapping). In other words, although the measured scene is isolated in the spatial domain, it is still continuous on each pixel along the temporal axis under a high-speed recording. When projecting N-step phase-shifting fringe patterns, it is required that N must be more than or equal to 4 to ensure that each period has four sampling points (In this paper, we adopt the nine-step phase-shifting). Once the sampling theorem in the temporal domain is not satisfied, the accuracy of the TFTP method will be affected. Therefore, for objects with complex dynamic distributions, the reconstruction accuracy of the TFTP method is between those of the FTP method and PMP method. In short, TFTP has a better performance on the dynamic measured scene and high-speed device can offer a better guarantee of the continuity in temporal domain.
- **A fast, low-cost and flexible structured light pattern sequence projector is presented.** In this paper, we presented a self-made mechanical projector to offer a better guarantee of the sampling theorem for the TFTP method. Our self-made projector generates sinusoidal fringe by the defocusing method and can reach the projection speed of thousands of frames per second. The signal part on the dics can accurately feedback the phase-shifting information and control the camera to capture the corresponding deformed pattern simultaneously. In addition, the dics is easy to change according to different measurement scenarios. In the measurement process, the measuring speed of the device is only limited by the shooting speed of the high-speed camera. Moreover, it is worth reminding that the sinusoidal feature generated from the defocused binary pattern will be affected by the defocusing degree, which is a common limitation of the binary defocusing method. So, if the measured height’s change exceeds the measurable depth range of the current binary defocusing, this proposed method will produce reconstruction error or even lead to failure.
- **The different spatial frequencies are adopted to match the measured process with different complexities.** It is a simple and effective method by introducing the reference plane to assist the absolute phase recovery of the 3-D wrapped phase distribution. According to the nature of the principle, the height change of the measured object must have one moment within the phase change of 2π corresponding to the height change range during the measurement. However, it is worth mentioning that the period of the projected sinusoidal fringe in the spatial domain does not affect the accuracy of the TFTP method (even the single-period sinusoidal fringe can be used). We can choose different spatial frequencies according to different measurement scenarios. For a special measured scene, in which the depth change at each moment in the measurement process is large, we can adopt the large period fringe projection to guarantee feasibility of 3-D phase unwrapping based on the reference plane.

## 5. Conclusions

In this paper, a reference plane is used to assist the 3-D phase unwrapping process. Combining it with the TFTP method, a new 3-D reconstructed result of isolated and abrupt objects can be obtained by every new deformed fringe pattern. So, it can successfully avoid motion-induced error in high-speed measurement. During the measurement process, one-dimensional Fourier transform, filtering and inverse Fourier transform are performed for each pixel along the time axis. Without band-pass filtering in 2-D spatial domain, the high frequency information of the object can be well preserved from being smoothed-out in the spatial domain. The known unwrappped phase of the reference plane is used to guide the phase unwrapping of one moment in the 3-D wrapped phase distribution, and then the 1-D phase unwrapping of each pixel along the time axis is carried out. For any pixel in the spatial domain, the phase extraction and phase unwrapping process are conducted independently, and the information of adjacent pixel points in the spatial domain is not refered. Therefore, it can be well used to measure the 3-D shape of a complex scene. Based on these ideas, a fast, low-cost and flexible structured light pattern sequence projector has been designed, which is capable of projection frequencies in the kHz level. Mainly limited by the employed cameras, our 3-D shape measuring system achieves reconstruction of an isolated and abrupt measured object at a rate of 297 Hz. 

## Supplementary Materials

The following are available online at https://www.mdpi.com/1424-8220/20/7/1808/s1.
